# “It isn’t Going to Last Forever!” The Effects of COVID-19 Pandemic on Older Adults

**DOI:** 10.1093/geroni/igab046.2293

**Published:** 2021-12-17

**Authors:** Chih-ling Liou

**Affiliations:** Kent State University, North Canton, Ohio, United States

## Abstract

The media puts a spotlight on older adults’ vulnerability to COVID-19 with limited consideration on how they view and cope with this crisis. This study is to give older adults voices to share their experiences of this pandemic. Data were collected using semi-structured interviews with 46 adults between the ages of 66 and 97 from the midwestern United States. Participants were asked to share how the pandemic affects them, their vision for the future, and how they cope during the pandemic. Although they worried about their health, felt isolated and missed seeing family and friends, most participants shared an optimistic view for the future. Some said that they are looking forward to receiving the vaccine, some believed that the effects of the pandemic are just temporary, and others compared that of the pandemic to the wars and other types of hardships which were much worse. Strategies for coping during the pandemic vary from spiritual practices to positive thinking, from exercises to new hobbies, and from calling family members to cutting down on the news. The results also show that the oldest-old and old-old participants seem to be better at regulating their negative effects compared to the young-old. One female participant in her 90s shared that she does not worry about the pandemic because there is nothing that she can do about it. Older adults’ adaptability during the COVID-19 pandemic should be better understood to reverse the image of their vulnerabilities and promote late-life coping during crises.

